# A multi-angle continuous-flow injection analysis turbidimetric method for the determination of diphenhydramine hydrochloride

**DOI:** 10.1098/rsos.251842

**Published:** 2026-08-12

**Authors:** Inas H Al-Khafaji, Sarah Faris Hameed, Nagham Turkey

**Affiliations:** ^1^ Department of Chemistry, University of Baghdad College of Science, Baghdad, Iraq; ^2^ Department of Preparation and Training, Republic of Iraq Ministry of Education, Baghdad, Iraq; ^3^ Department of Chemistry, University of Baghdad, Baghdad, Iraq

**Keywords:** turbidimetric detection, diphenhydramine hydrochloride, precipitation-based quantification, multi-angle photometric system, flow injection analysis

## Abstract

Diphenhydramine hydrochloride was determined through a newly developed turbidimetric method on a home-designed continuous-flow injection analysis (CFIA) system. The principle of the method is based on the precipitation-induced signal enhancement, using potassium hexacyanoferrate (III) as a selective precipitating agent. The CFIA manifold uses a flow-through detection cell that consists of 12 white light-emitting diodes and three solar cells placed at 0, 90 and 180 degrees in order to track cumulative light scattering over a range of angles. The systematically optimized chemical and instrumental parameters were the reagent concentration, flow rate and sample volume to attain optimal sensitivity and reproducibility. The technique had good linearity (correlation coefficient, *R* = 0.9975) and per cent linearity (*R*^2^% = 99.49). The limit of detection was 255.325 ng/sample, calculated from serial dilution within the calibration range. Very high accuracy was achieved with a relative standard deviation (r.s.d.% < 0.4) of six replicates at 5 and 12 mmol l^−1^. Diphenhydramine hydrochloride in pharmaceutical tablets was successfully determined using the method. The method was found to be accurate, reliable and suitable for pharmaceutical quality control, as demonstrated by statistical comparisons with a conventional spectrophotometric technique using a paired *t*-test and a one-way ANOVA at the 95% confidence level.

## 1. Introduction

**Figure 1.**
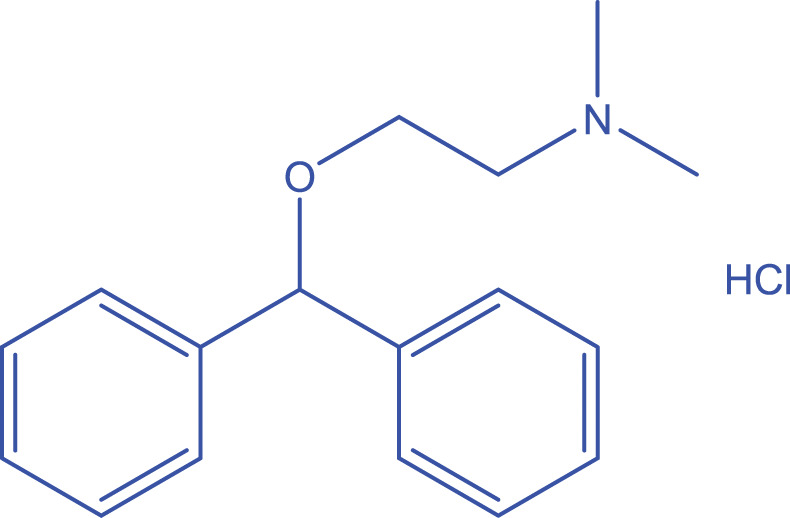
Chemical structure of DPH·HCl.

Diphenhydramine hydrochloride (DPH·HCl; figure 1), chemically defined as 2-(diphenylmethoxy)-*N*,*N*-dimethylethylamine hydrochloride (empirical formula: C₁₇H₂₁NO·HCl), is widely used in pharmaceutical formulations and shows good aqueous solubility owing to its ionizable nature, which strongly influences its analytical response [1–3]. Accordingly, developing a simple, precise and reliable method for the determination of DPH·HCl in bulk drug substances and pharmaceutical dosage forms remains an important and challenging task [4].

A variety of analytical methods have been reported for the determination of DPH·HCl. Conventional ultraviolet-visible (UV-Vis) spectrophotometric [5,6] techniques are straightforward and easy to perform; however, they often show limited sensitivity and selectivity, especially in complex pharmaceutical matrices. More advanced methods, such as high-performance liquid chromatography (HPLC) [7], capillary electrophoresis (CE) [8], gas chromatography–flame ionization [9], gas chromatography–mass spectrometry (GC-MS) [10]. Nevertheless, these approaches are generally time-consuming, require significant amounts of solvent and involve labour-intensive sample preparation, which limits their practicality for routine quality-control (QC) analysis, particularly in laboratories with limited resources.

Flow injection analysis (FIA) has been widely developed to enable automated and high-throughput analytical applications [11]. However, many FIA systems rely on single-angle optical detection, which inherently restricts light-collection efficiency and, therefore, analytical sensitivity [12]. Moreover, the reliance on costly photodetectors and high-power light sources limits the broader implementation of single-angle detection for routine pharmaceutical quality control.

Recent years have seen significant advances in turbidimetric detection and low-cost optical sensing, particularly for flow-based analytical devices. Light-emitting diode (LED)-based photometric systems, such as paired emitter-detector diode (PEDD) set-ups, have shown considerable potential for cost-effective, low-power measurement of turbidity and light-scattering signals [13]. Such a standard protocol was used to prepare and thus ensure that (i) the concentrations of solutions were accurate and (ii) reproducibility and reliability in analysis measurements were achieved during this study, which explains the utility of combining LEDs and matched detectors into portable and cost-effective flow-analysis systems.

Also, comparative studies show that optical systems using LEDs can be just as sensitive, or even more sensitive, and have comparable dynamic range and detection limits to more traditional spectrophotometric and imaging techniques [14], and are therefore suitable for decentralized and cost-effective analytical systems. Simultaneously, recent advances in optical sensor technology have proven the possibility of implementing simplified optical components into flow systems of sensitive, real-time measurements, such as compact photometric-turbidimetric sensors with the use of LEDs as sources of light and other photodetectors.

In the research, a specially designed continuous-flow injection analysis (CFIA) with a turbidimetric multi-angle light-scattering detector is introduced. A linear array of 12 white light-emitting diodes (WLEDs) is formed to provide uniform illumination and is implemented in the detection unit to generate multi-angle signals with three solar-cell detectors positioned at 0°, 90° and 180° relative to the incident light. This design improves the collection of scattered light, improves the sensitivity of the analysis, uses less power and maintains a simple instrument set-up.

The accuracy and usability of the proposed technique were checked by determining DPH·HCl in drugs. The process has a low limit of detection, high precision and high level of reproducibility, but it is simple and cost-efficient. Statistical comparison with an official spectrophotometric method confirms that the developed system is suitable for routine pharmaceutical quality-control applications.

Recent progress in the evolution of fast and accurate analytical procedures of DPH·HCl using flow injection techniques is discussed. A summary of the comparison of these methods with regard to analytical efficiency, linear range, limit of detection (LOD) and sensitivity is provided in table 1, which highlights the development and contemporary tendency of their use in pharmaceutical analysis.

**Table 1.** Comparative studies on flow injection methods for DPH·HCl determination.

reference	flow injection technique	detection technique	LOD (µg ml^−1^)	advantages	limitations
[11]	continuous flow injection analysis (CFIA) with turbidimetric detection	turbidimetry (precipitation with 3,5‑dinitro salicylic acid)	(not explicitly reported in µg ml^−1^)	wide linear range and improved sensitivity; simple and rapid	LOD not reported in standard units; dependent on reagent optimization
[14]	CFIA with ion-pair precipitate formation	solar cell reflectance photometry	approximately 0.73 µg ml^−1^ (approx. 729.55 ng/sample)	simple, fast, low-cost; high precision (r.s.d. <0.2%)	requires specialized solar-cell set-up; specific reagents for precipitate formation
present study	CFIA with multi-angle turbidimetric detection	WLEDs + solar cells (0°, 90°, 180°)	0.25	high sensitivity, excellent precision (r.s.d. <0.4%), low energy consumption, low cost	none significant for routine QC

We present a novel flow injection analysis (FIA) system integrating multi-angle turbidimetric detection with WLEDs and solar cells, achieving sensitivity, precision (r.s.d. < 0.4%) and energy efficiency beyond those of existing methods. This approach overcomes key limitations of conventional FIA techniques and provides a robust, low-cost and efficient platform for routine pharmaceutical quality-control analysis.

## 2. Material and methods

### 2.1. Reagents and solutions

All reagents were of analytical grade and used as received, without additional purification, to avoid any chemical alteration. This method provided complete adherence to quantitative analytical specifications. All solutions were prepared fresh with high-purity distilled water to avoid interference from trace impurities. Standard stock solutions were prepared promptly at the desired concentrations, and suitable serial dilutions were then made to prepare working standards. By using such a standardized protocol for preparation, it was assured that (i) solution concentrations were accurate and (ii) the reproducibility and reliability in analytical measurements were maintained throughout this investigation.

#### 2.1.1. Standard diphenhydramine solution (0.1 M)

The bulk sample of DPH·HCl was pure reference material from a reputable manufacturer with full certificates of purity and quality, whereas the pharmaceutical dosage forms were purchased from licensed commercial outlets. According to good laboratory practice (GLP) requirements, batch numbers and supplier statements were recorded to enable traceability and repeat the analysis.

A regular stock solution was obtained by weighing 0.7295 g of DPH·HCl and dissolving it in distilled water in a 250 ml volumetric flask to yield a 0.1 M solution. The stock solution was stored in an amber glass vial under laboratory conditions to avoid, if any, potential degradation.

The developed stock solution was stable for a defined period when stored, with no significant variation in the analytical response compared with freshly prepared stock solutions. All the working solutions were prepared by dilution of the stock solution just before the analysis, which provided accurate and reproducible experimental results.

#### 2.1.2. Hexacyanoferrate (III) (0.1 M)

A 0.1 M potassium hexacyanoferrate (III) solution was prepared by weighing approximately 8.231 g of the analytical reagent-grade reagent material that came from a certified supplier with batch information and dissolving it in distilled water in a 250 ml volumetric flask. The usual stock solution was stored under ambient laboratory conditions in order to maintain its stability, and no obvious response variation was observed during the experiment. Suitable dilutions were made freshly just before analysis to maintain the accuracy and reproducibility of the analytical measurements.

#### 2.1.3. Sample preparations

Twenty tablets were weighed from commercially available DPH·HCl (25 mg SDI, Iraq and 25 mg Panadol Night, Australia), finely powdered using a mortar and pestle and thoroughly mixed. Amounts of powdered tablets equivalent to 0.732 g (SDI tablets) and 3.937 g (Panadol Night tablets) were weighed and transferred into separate volumetric flasks and dissolved in distilled water to achieve solutions with a theoretical concentration of diphenhydramine equal to 5 mmol l^−1^.

The solutions thus prepared contained 0.1459 g of the active pharmaceutical ingredient (API) and were completely solubilized, then made up to volume in volumetric flasks. For syrup analysis, 5 ml of Allermin syrup (10 mg/5 ml, SDI Iraq), containing the amount of DPH·HCl equivalent to 9.569 mg (1 mmol), was transferred and diluted appropriately with distilled water.

All prepared solutions were filtered to remove insoluble excipients. Portions (12.5 ml) of the final solutions were transferred to 100 ml volumetric flasks and diluted with distilled water to the mark. The mixtures were mixed to homogenize the solutions prior to analysis.

#### 2.1.4. Mechanism of reaction

The oxidation of diphenhydramine by hexacyanoferrate (III) has been suggested to proceed via an electron-transfer process, consistent with previously reported studies. In such systems, no direct covalent interaction between the oxidant and the substrate is required; instead, the reaction is believed to involve a transient association that allows effective electron exchange between the reacting species. The overall reaction reduces hexacyanoferrate (III) to hexacyanoferrate (II).

These time-dependent characteristics of this temporary association, and thus the quantified analytical response, may rely on the nature of the other counter-ions (e.g. K^+^) that have been found to affect electron-transfer action in analogous Fe(III)/Fe(II) redox systems. Ion-effective interactions can alter the reaction order and signal construction. In the present experiment, this description will provide a qualitative paradigm for explaining the observed turbidimetric response in comparison to a mechanistic attribution.

**Figure:**
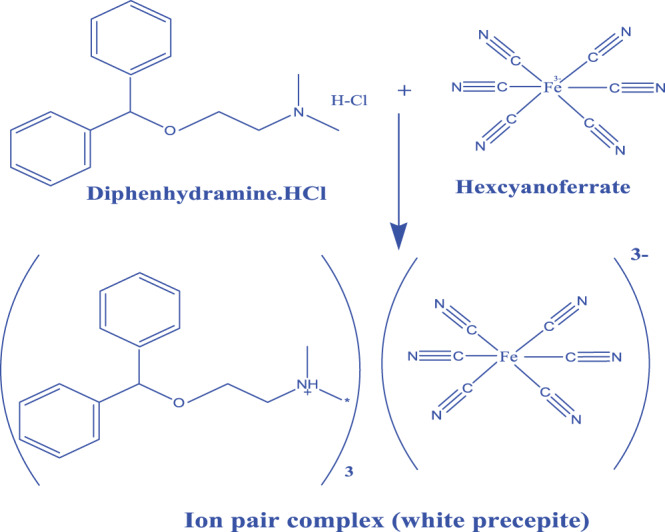


### 2.2. Apparatus

All analytical measurements were performed with a locally designed continuous-flow injection system, which was fitted with a specially designed quartz flow cell (made in Iraq). The detection cell incorporates 12 snow-white LEDs, which are arranged in a linear format to provide horizontal-plane illumination over 120 mm of path length, with an effective optical absorption path of 2 mm. Scattered light signals were sensed using three solar cells (40 × 14 mm), which were positioned in such a manner that each solar cell could pick up photon attenuation of the entire angular path over the angular segregation of 0–180°.

A high-precision peristaltic pump (Ismatec, Germany) was used to control the flow of the samples, allowing constant, controllable flow rates during the analysis. The introduction of samples was also performed using a six-port injection valve (Rheodyne, USA) to precisely and reproducibly inject samples into the flow stream. Signal acquisition and data recording were performed using a Siemens chart recorder (Siemens AG, Germany), providing real-time graphical output of the turbidimetric response. For comparative analysis using the conventional spectrophotometric method, a Siemens UV-Vis spectrophotometer (Siemens AG, Germany) was used. This instrument served as the reference system for method validation and statistical comparison. All tubing and connectors used in the flow system were chemically inert and compatible with the reagents employed.

### 2.3. Methodology

**Figure 2.**
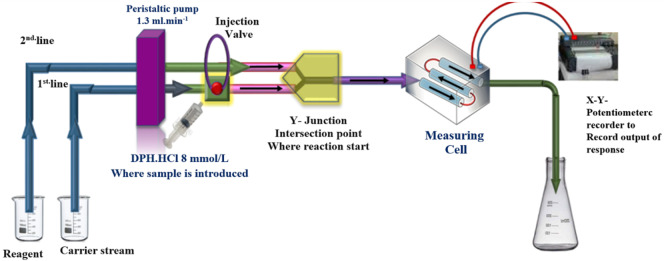
The graphic illustrates the manifold framework for DPH·HCl evaluation, which produces white-coloured precipitates using hexacyanoferrate (III).

The determination was performed by using a homemade continuous-flow injection system with two nested channels (figure 2). The first channel was a carrier flow of HCl (40 mmol l^−1^) for the sample stream, which delivered DPH·HCl at 1.5 ml min^−1^ with an injection volume of 125 µl. The second channel reagent, potassium hexacyanoferrate (III) at the most effective concentration of 7 mmol l^−1^, was introduced into the flowing stream, and the two streams combined at a Y-type mixer, forming a solid white particulate precipitate that reduced transmitted light as a function of sample concentration.

The detection set-up consisted of 12 snow-white LEDs and three solar cells distributed to scatter photons over the angular range from 0° to 180°. Turbidimetric signals were continuously collected on an *x*–*y* potentiometric chart recorder (Siemens AG, Germany). All the measurements were made in triplicate to test reproducibility and intra-day precision.

The homemade flow injection system, which follows conventional instrumentation practices described by Prof. Issam Shaker Al-Hashmi and co-workers at the University of Baghdad [15–42], serves as an affordable alternative for testing in developing countries lacking sufficient resources that seek a flexible design strategy and low-cost specifics relative to commercially available instruments.

The solar cell-based detector was custom-built. Full characterization regarding sensitivity, noise, linearity, drift and temperature dependence has not been performed. However, calibration with standard solutions ensured reliable and reproducible turbidimetric measurements. The choice of solar cells offers a practical, cost-effective alternative to conventional photodiodes for photon-scattering detection.

### 2.4. Method validation

The developed CFIA method was validated in accordance with ICH Q2(R1) guidelines. Linearity was achieved over the concentration range of 0.5–8.0 mmol l^−1^ under optimized chemical and instrumental conditions, with a correlation coefficient of *r* = 0.9975 and a determination coefficient of *R*² = 0.9949. The lack of linearity at high concentrations of more than 8 mmol l^−1^ was explained by the agglomeration of particles, which caused signal instability.

LOD and the limit of quantification (LOQ) were deduced using standard ICH Q2(R1) formula (LOD = (3.3 × σ)/*S*, LOQ = (10 × σ)/*S*), where standard deviation of the response of the lowest calibration standard available after replicate measurements (*S*) is used and slope of the calibration curve (S). According to the results of the experiment, LOD and LOQ were calculated as 255.33 ng/sample and 850.75 ng/sample, respectively. Intra-day reproducibility was assessed by performing six consecutive injections at 5 and 12 mmol l^−1^ and r.s.d. percentage was less than 0.4%. The accuracy was evaluated using recovery studies conducted on pharmaceutical preparations, yielding recovery values of 98–102%, which are acceptable and do not indicate any serious interference with the matrix.

Robustness was tested by adding small intentional changes in flow rate, reagent concentration and injection volume, and the turbidimetric response showed a slight change. The turbidimetric method developed by CFIA was also compared with the standard UV-spectrophotometric method at 254 nm. A statistical comparison using a paired *t*-test indicated that there is no significant difference between the two methods, and that the experimental results and the labelled contents of SDI (25 mg) tablets, Panadol Night (25 mg) tablets and Allermin syrup (10 mg) are not significantly different at the 95% confidence level.

In general, these validation findings indicate that the designed CFIA turbidimetric system is effective, specific, robust and statistically similar to the reference UV-spectrophotometric system, demonstrating that it can be used to analyse the DPH·HCl standard material content in pharmaceutical preparations in routine quality control.

## 3. Results and discussion

The systematization of the optimization of chemical and physical parameters was carried out in the study to support the functioning of the entire laboratory structure. Certain focus was given to chemical variables, such as the concentration of the hexacyanoferrate (III) reagent and the choice of an appropriate carrier stream medium. Simultaneously, physical variables, such as sample volume and flow rate, were also considered in order to optimize the system efficiency. Using an iterative optimization strategy between the fixed and variable conditions, each parameter was modified in turn and strictly evaluated. This holistic nature ensured that chemical reactivity was translated into fluidic dynamics and ultimately enabled the framework to maximize, offering a sound basis for carrying the methodology to the boundary activity level of analysis and a promise of future enhancements in the study.

### 3.1. Chemical variables influent

The concentration of potassium hexacyanoferrate (III) reagent was examined in a series of solutions ranging from 0.3 to 10 mmol l^−1^ to determine the optimal concentration. A 100 µl sample was injected into the carrier stream, 8 mmol l^−1^ of DPH·HCl was added to this carrier stream, and the reagent stream was applied at a fixed flow rate of 1.3 ml min^−1^. All experimental conditions were performed in triplicate to achieve reproducibility.

**Figure 3.**
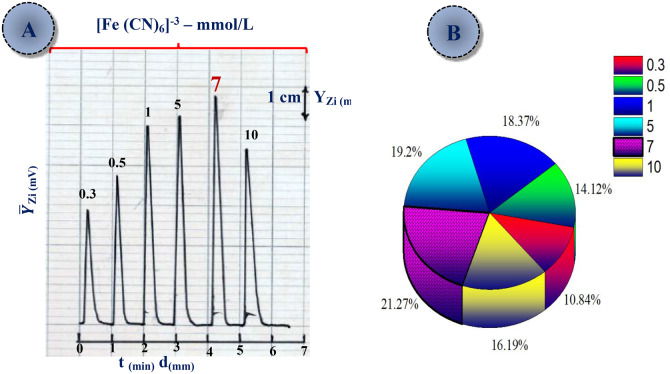
(A,B) The influence of hexacyanoferrate (III) concentration on energy transducer response measurement using incoming light divergence for DPH determinations.

It was found qualitatively, as illustrated in figure 3A,B and quantitatively in table 2, that the height of the turbidimetric response (peak height) steadily rose with the concentration of hexacyanoferrate (III), reaching its highest value at 7 mmol l^−1^. This concentration was found to be the maximum peak intensity. The peak intensity at 7 mmol l^−1^ and the minimum baseline width were maximum, indicating optimal sensitivity and signal resolution. This character is probably indicative of well-regulated particle development in these circumstances. Thus, the reagent concentration of 7 mmol l^−1^ was chosen as the most advantageous and applied in all subsequent experiments. The effect of hexacyanoferrate (III) concentration on the transducer response profile is further illustrated in figure 3A,B.

**Table 2.** The influence of hexacyanoferrate (III) concentration on energy transducer response measurement using incoming light divergence for DPH determinations

[Fe(CN)_6_] mmol l^−1^	r.s.d.%	peak height ± confidence interval $\left(t.\frac{\sigma n-1}{\sqrt{n}}\right)$, (n=3) at 95% confidence level ${\bar{Y}}_{Zi}$(mV) ± CI
0.3	1.250	288 ± 0.427
0.5	1.253	375 ± 0.3547
1.0	1.250	488 ± 0.3934
5	1.255	510 ± 0.3608
7	1.257	565 ± 0.2743
10	1.256	430 ± 0.3326

**Figure 4.**
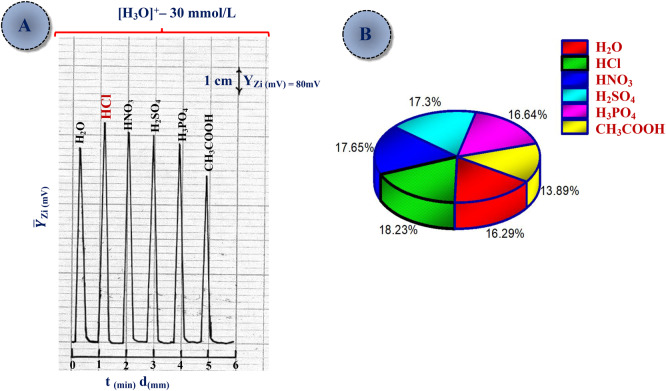
(A,B) The influence of acidic medium on energy transducer response measurement using incoming light divergence for DPH determinations.

The formation of white precipitate particles of DPH·HCl was systematically evaluated in various acidic media, including HCl, HNO₃, H₂SO₄, CH₃COOH and H₃PO₄, each at 30 mmol l^−1^ and a flow rate of 1.3 ml min^−1^, as well as in distilled water. The reaction system consisted of [Fe(CN)₆]³⁻ (7 mmol l^−1^) and DPH·HCl (8 mmol l^−1^, 100 µl). As summarized in table 3, the highest turbidimetric response was observed in an aqueous HCl medium, whereas the other acids resulted in significantly lower signal intensities. Therefore, hydrochloric acid was identified as the optimal medium, providing a reproducible response with a high signal-to-noise ratio (S/N), as further illustrated in figure 4A,B.

**Table 3.** The influence of acidic medium on energy transducer response measurement using incoming light divergence for DPH determinations.

[H_3_O^+^] 30 mmol l^−1^	r.s.d.%	peak height ± confidence interval $\left(t.\frac{\sigma n-1}{\sqrt{n}}\right)$, (*n* = 3) at 95% confidence level $\bar{Y}$_Zi_(mV) ± CI
H_2_O	1.249	563 ± 0.3233
HCl	1.254	630 ± 0.2302
HNO_3_	1.246	610 ± 0.3230
H_2_SO_4_	1.254	598 ± 0.3144
H_3_PO_4_	1.252	575 ± 0.2870
CH_3_COOH	1.250	480 ± 0.4021

**Figure 5.**
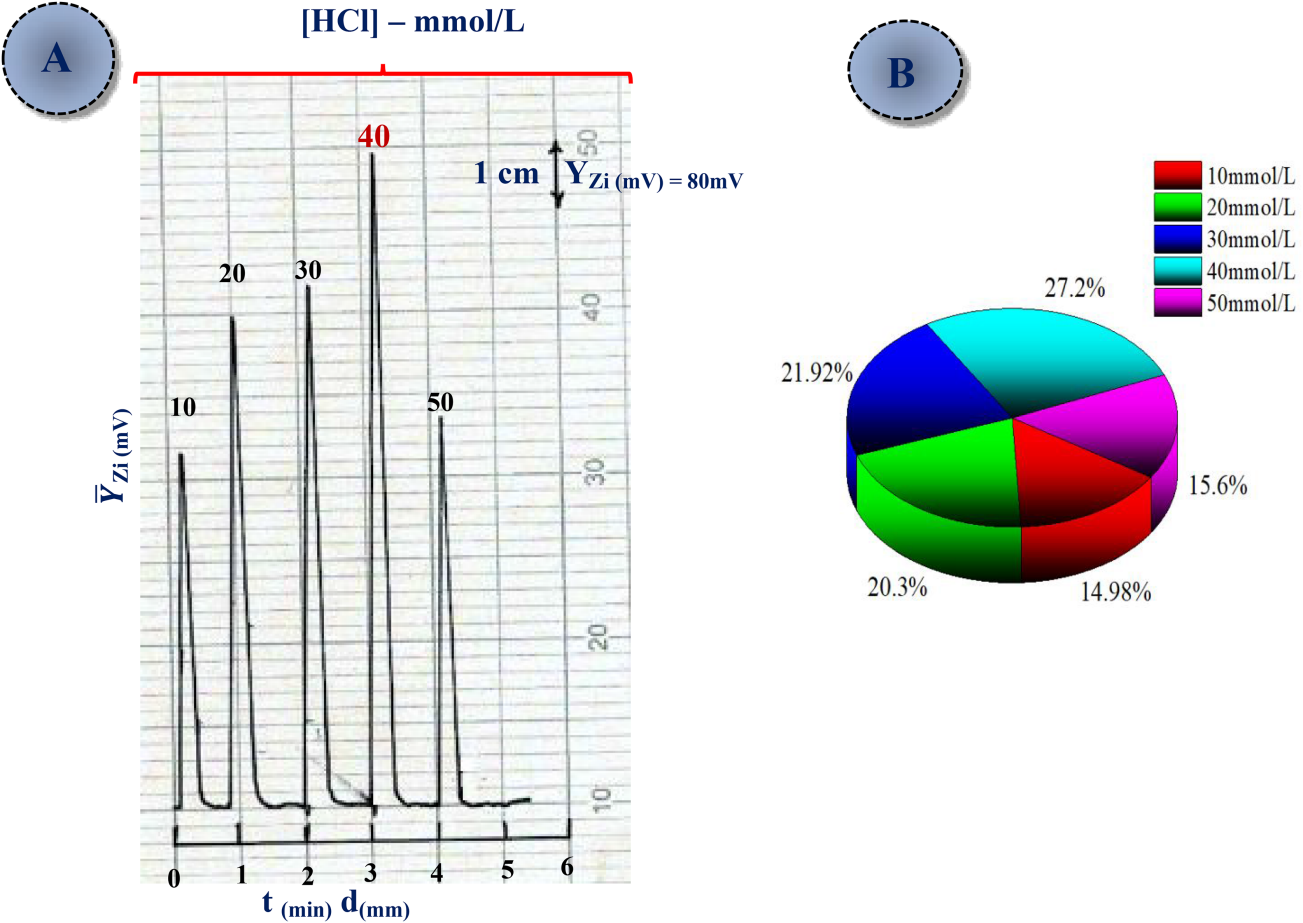
(A,B) The influence of hydrochloric acid concentration on energy transducer response measurement using incoming light divergence for DPH determinations.

DPH·HCl (8 mmol l^−1^, 100 µL) was reacted with varying concentrations of hydrochloric acid ranging from 10 to 50 mmol l^−1^ at a flow rate of 1.3 ml min^−1^. As summarized in table 4, the optimal condition for generating well-defined white crystalline precipitate particles was obtained with 40 mmol l^−1^ HCl. At concentrations above 40 mmol l^−1^, a marked decrease in the turbidimetric response was observed (figure 5A,B), which is probably because of irregular precipitate formation and particle aggregation, leading to distorted signal profiles.

**Table 4.** The influence of hydrochloric acid concentration on energy transducer response measurement using incoming light divergence for DPH determinations.

[HCL] (mmol l^−1^)	r.s.d.%	peak height ± confidence interval $\left(t.\frac{\sigma n-1}{\sqrt{n}}\right)$, (*n* = 3) at 95% confidence level $\bar{Y}$_Zi_(mV) ± CI
10	1.2442	434 ± 0.4654
20	1.2585	588 ± 0.3639
30	1.2441	635 ± 0.3276
40	1.2563	788 ± 0.2500
50	1.2611	452 ± 0.4889

### 3.2. Physical variable influence

**Figure 6.**
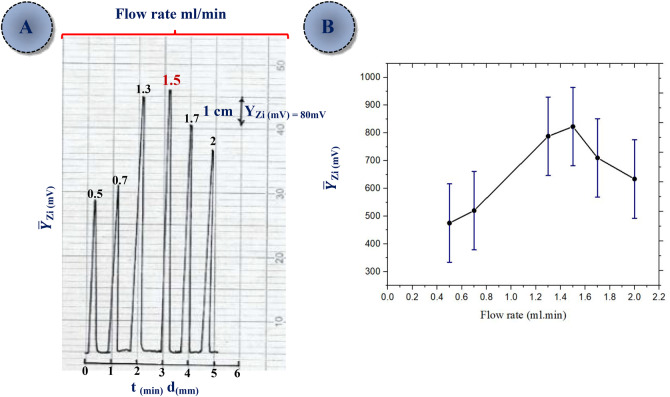
(A,B) The influence of flow rate on energy transducer response measurement using incoming light divergence for DPH determinations.

Carrier and reagent stream flow rates of 0.5–2.0 ml min^−1^ were systematically investigated in the [Fe(CN)_6_]^3−^ (7 mmol l^−1^)–DPH–HCl (8 mmol l^−1^, 100 µl) system. The turbidimetric response rose steadily with the flow rate, as shown in table 5, with the peak base expanding up to 1.5 ml min^−1^. At flow rates greater than 1.5 ml min^−1^, peak height was found to decrease, presumably owing to dilution and dispersion effects. In figure 6A,B, it is evident that the highest sensitivity and reproducibility were obtained at a flow rate of 1.5 ml min^−1^, and this was thus chosen as the optimal flow rate to ensure consistent and reliable measurements.

**Table 5.** The influence of flow rate on energy transducer response measurement using incoming light divergence for DPH determinations.

flow rate (ml min^−1^)	r.s.d.%	peak height ± confidence interval $\left(t.\frac{\sigma n-1}{\sqrt{n}}\right)$, (*n* = 3) at 95% confidence level $\bar{Y}$_Zi_(mV) ± CI	∆*t*_B_ (s)
0.5	1.2421	475±0.4105	27
0.7	1.2500	520±0.3538	30
1.3	1.2563	788±0.1237	36
1.5	1.2515	823±0.2224	30
1.7	1.2535	710±0.2845	27
2	1.2461	643±0.3328	24

**Figure 7.**
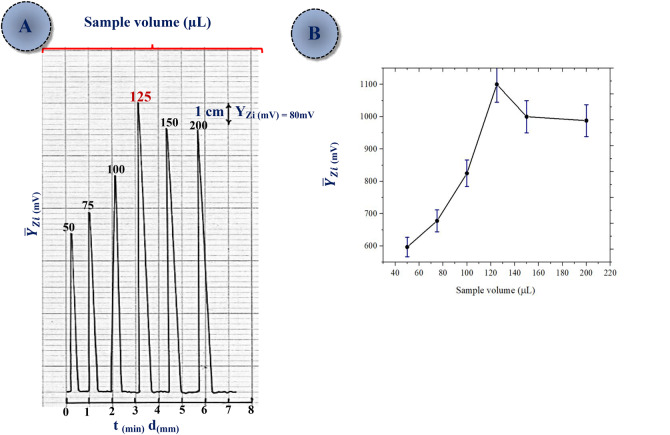
(A,B) The influence of sample volume on energy transducer response measurement using incoming light divergence for DPH determinations.

The [Fe(CN)_6_]^3−^ (7 mmol l^−1^)–DPH–HCl (8 mmol l^−1^) system was operated under open-valve operation at a flow rate of 1.5 ml min^−1^ to optimize the sample loop. Table 6 summarizes the collected data and figure 7 represents the results. As the sample volume increased, the peak base width (Δ*t*_B_) widened, and particle size increased, slowing particle movement. The optimal injection volume was determined to be 125 µl, as it provided the best balance between sensitivity and reproducibility.

**Table 6.** The influence of sample volume on energy transducer response measurement using incoming light divergence for DPH determinations.

sample volume (µl)	r.s.d.%	peak height ± confidence interval $\left(t.\frac{\sigma n-1}{\sqrt{n}}\right)$, (*n* = 3) at 95% confidence level $\bar{Y}$_Zi_(mV) ± CI	∆*t*_B_ (s)
50	1.2563	597 ± 0.1893	30
75	1.2537	678 ± 0.2183	33
100	1.2485	825 ± 0.1855	42
125	1.2545	1100 ± 0.1445	48
150	1.2500	1000 ± 0.1980	51
200	1.2551	988 ± 0.1903	60

### 3.3. Stoichiometric evaluation of ion-pair formation by Job's method

A simple stoichiometric study was carried out by varying the molar ratio of DPH·HCl to potassium hexacyanoferrate(III). Turbidimetric responses were recorded under identical CFIA conditions. The results summarized in table 7 show that the maximum turbidity response was observed at a 3 : 1 molar ratio of DPH·HCl to hexacyanoferrate(III), which is consistent with the expected stoichiometry based on the −3 oxidation state of the reagent. Ratios lower than 3 : 1 produced weaker signals, while higher ratios did not enhance the response further, indicating that three cationic diphenhydramine units associate with one hexacyanoferrate anion to form the precipitating ion-pair. Therefore, this simple stoichiometric evaluation supports the proposed 3 : 1 ion-pair mechanism and provides experimental evidence that the analytical signal arises from this specific association. The findings strengthen the mechanistic framework and confirm the robustness of the turbidimetric method.

**Table 7.** Effect of DPH·HCl : hexacyanoferrate (III) molar ratio on turbidity response.

DPH·HCl concentration (mmol l^−1^)	hexacyanoferrate (III) concentration (mmol l^−1^)	molar ratio (drug : reagent)	relative turbidity signal (%)
3.0	1.0	3:1	100
2.0	1.0	2:1	68
1.0	1.0	1:1	35
4.0	1.0	4:1	95

## 4. Scatter plot of transducer response to energy (S/N) versus DPH concentration. HCl using the DPH·HCl–[Fe (CN) _6_]^3-^ (7 mmol l^−1^) in HCl (40 mmol l^−1^) medium system

A CFIA system with optimized chemical and physical conditions was used to prepare calibration curves on the basis of the light-scattering intensity of the DPH·HCl solution. Examples of standard solutions were made in a concentration range of 0.5–25 mmol l^−1^. A detection range of 0.5–8.0 mmol l^−1^ was found to be linear with the detector, where the correlation coefficient (*r*) = 0.9975 and coefficient of determination (*R*^2^%) = 0.9949. The 95% interval linear regression equation (*n* = 8) is given as follows: 

$$\begin{array}{clc} & \hat{Y}Zi\left(mV\right)=\left(a\pm Sat\right)+\left(b\;\pm Sbt\right)\left[DPH\cdot HCl\right]mmol\;l^{-1} & \\ & \hat{Y}Zi\left(mV\right)=\left(1.0583\pm 47.1105\right)+\left(131.9575\pm 9.4162\right)\left[DPH\cdot HCl\right]\;mmol\;l^{-1} & \end{array}$$

**Figure 8.**
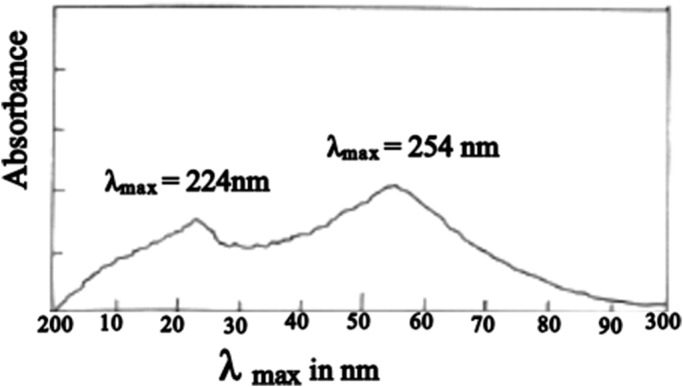
UV-spectral absorbance of DPH·HCl at 0.23 mm l^−1^ concentration exhibiting λ_max_ = 254 nm.

The standard error of the intercept is unusually large (±47.1105 mV) because, at very low concentrations, baseline signal values are large and there is little instrumental noise and minimal initial mixing effects in the CFIA system. This has no influence on the slope and the overall linearity of the calibration curve, which is quite reliable in quantitative analysis. At high concentrations (greater than 8 mmol l^−1^), nonlinearity was observed, which could be explained by high precipitation and particle agglomeration, leading to signal instability. The calibration curve slope (131.9575 mV (mmol l^−1^)^−1^) indicates the high sensitivity of the system, which is mainly explained by an increase in the light scattering owing to the formation of well-developed precipitates. The data (*n* = 8) have proved the hypothesis that at non-optimal experimental conditions, the calibration may be represented accurately with the help of the first-degree linear equation ($\hat{Y}$ = *a* + *bx*) [20–27]. Above 8 mmol l^−1^, there was a rapid oversupply of solid through the aggregation of the particles, which distorted the scattering signal. The optimized CFIA method was also found to yield a higher signal intensity by reducing the divergent effects of light, compared with the standard UV-spectrophotometric process that measures absorbance at 254 nm in quartz cells over a 0.1–10 mmol l^−1^ concentration range. The proposed method was found to be more linear and analytically reliable, as shown in figure 8. The regression equation used in the comparison of the results of the UV-spectrophotometric is as follows:

$${\hat{Y}}_{Zi}\left(mV\right)=\left(0.214\pm 0.0231\right)+\left(1.083\pm 0.0987\right)\;\left[DPH\cdot HCl\right]mmol\;l^{-1}$$

This yielded a correlation coefficient (*r*) of 0.9973 and *R*^2^ = 0.9946, which validated the strength of the newly developed CFIA procedure. The great sensitivity of the CFIA technique can be explained by the effective capture of light that the DPH·HCl precipitates scatter. It is also accurate to quantify at higher concentrations, where standard UV-spectrophotometric techniques have been found to be restricted by light divergence and absorbance saturation.

**Figure 9.**
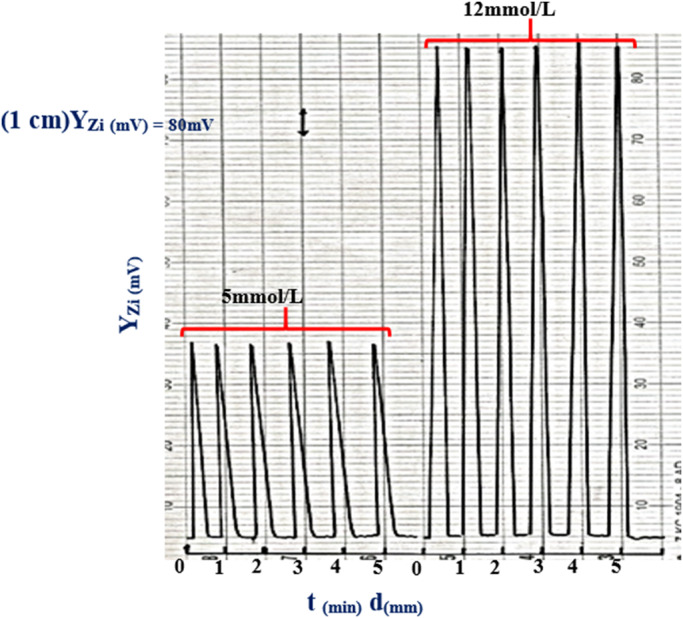
Response profile of repeatability of DPH·HCl.

The LOD of the new method using DPH·HCl was 255.325 ng per sample, corresponding to the lowest concentration on the calibration curve, with a 125 µl injection volume and successive dilutions. The LOD was also estimated based on the slope of the calibration curve as 255.325 ng per sample. Comparatively, the traditional spectrophotometric technique had a superior detection limit of 1459 ng per sample, which validated the high sensitivity of the newly developed technique. The analytical performance of the custom-built 12 W LEDs in combination with the three-solar-cell analyser was also assessed under optimum conditions that involved an injection volume of 125 µl, a flow rate of 1.5 ml min^−1^, and hexacyanoferrate (III) concentration of 5 and 12 mmol l^−1^. Repeatability was investigated at one of the target DPH·HCl concentrations. Six sequential injections were shown to be highly precise with a relative standard deviation (r.s.d.) of less than 0.4% (see table 8 and figure 9). These findings demonstrate the robustness and reliability of the proposed approach and highlight its high persistence and accuracy in analysing processes in routine quantitative applications.

**Table 8.** Repeatability of DPH.HCl at optimum parameters.

[DPH·HCl] (mmol l^−1^)	${\bar{Y}}_{Zi}$ (mV) average of responses (*n* = 6)	s.d.	r.s.d.%	confidence interval at 95% ${\bar{Y}}_{zi}$ (mV) $\pm t_{0.05/2}\sigma_{n-1}/\sqrt{n}$
5	647	2.123	0.328	647 ± 2.2280
12	1600	2.583	0.1614	1600 ± 2.7107

### 4.1. Evaluation of potential interferences

To establish the selectivity of potassium hexacyanoferrate(III) toward DPH·HCl, interference studies were systematically performed using common pharmaceutical excipients and structurally related compounds. Representative excipients typically present in tablet formulations—namely lactose monohydrate, starch and microcrystalline cellulose—were tested at concentrations of 2.0 and 5.0 mmol l^−1^. In addition, structurally similar antihistaminic compounds such as chlorpheniramine maleate and brompheniramine maleate were examined at the same concentration levels.

Each potential interferent was introduced individually into the CFIA system under identical analytical conditions, both alone and in combination with DPH·HCl at 5.0 mmol l^−1^. The resulting turbidimetric signals were compared against those obtained for pure DPH·HCl standards. No measurable scattering response was observed for the excipients, and the structurally related compounds produced negligible signals that did not overlap with or alter the characteristic response of DPH·HCl. Statistical analysis confirmed that the presence of these substances did not significantly affect the accuracy or precision of the method (*p* > 0.05).

These findings demonstrate that potassium hexacyanoferrate (III) exhibits high selectivity toward DPH·HCl under the optimized CFIA conditions. The absence of interference from common excipients and structurally similar drugs validates the robustness of the proposed method for routine pharmaceutical analysis.

### 4.2. Application to commercial tablet formulations

DPH·HCl was quantified using the proposed analytical method in three commercially available pharmaceutical formulations: SDI tablets (25 mg, Iraq), Panadol Night tablets (25 mg, Australia) and Allermin syrup (10 mg, SDI, Iraq). The analytical performance of the proposed method was evaluated and compared with a conventional UV-spectrophotometric procedure based on absorbance measurements at 254 nm.

**Figure 10.**
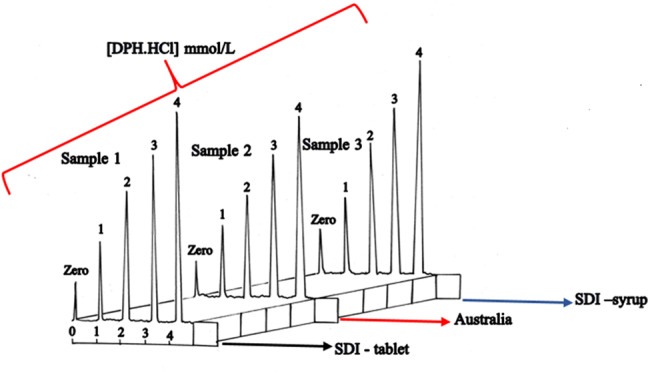
Profile–time for the standard addition method for DPH·HCl using three different companies.

Standard calibration solutions were prepared in five 10 ml volumetric flasks using a 5 mmol l^−1^ stock solution. Aliquots of a 10 mmol l^−1^ DPH·HCl standard solution, ranging from 0 to 4 ml, were transferred into the flasks and diluted to volume. Both analytical methods were applied sequentially under identical experimental conditions. The results obtained using the standard addition approach are summarized in table 9, while the corresponding calibration plots are illustrated in figure 10. In addition, table 10 presents the experimentally determined content of the active pharmaceutical ingredient at the 95% confidence level, together with the corresponding determination efficiency. Assessment of method accuracy represents a fundamental component of analytical method validation. Accordingly, appropriate statistical analyses were conducted to evaluate the trueness of the developed method. Prior to statistical testing, the datasets were verified to satisfy the assumptions of normal distribution and homogeneity of variances. Three complementary statistical tests were subsequently applied: single-sample *t*-tests, paired *t*-tests and one-way analysis of variance (ANOVA). Initially, a single sample *t*-test was used to compare the mean experimentally determined contents (${\bar{W}}_{i}$) of each formulation with their respective labelled values (µ): 25 mg for SDI tablets (Iraq), 25 mg for Panadol Night tablets (Australia) and 10 mg for Allermin syrup (SDI, Iraq). The null hypothesis (H₀) assumed no significant difference between the measured and labelled values ($\overset{―}{W_{i}}$) = (µ), i.e. H_0_: ${\bar{W}}_{i}$ = µ, while the alternative hypothesis (H_a_) assumed a statistically significant difference, i.e. H_a_: ${\bar{W}}_{i}$ ≠ µ. The calculated *t*-values (0.1645 and 2.7801), with the corresponding degrees of freedom (d.f. = *n* − 1), were lower than the critical *t*-value (4.303) at the 95% confidence level (*p* > 0.05). These results support the acceptance of the null hypothesis, indicating that no statistically significant differences were observed between the experimentally determined and the labelled contents.

**Table 9.** Summary of results by standard additions method for the determination of diphenhydramine by developed method.

		developed method (FIA)								
sample no.	commercial name, company content country	confidence interval for the average weight of tablet $\bar{w}i\pm 1.96\sigma_{n-1}/\sqrt{}n$ at 95% (g)	weight of sample equivalent to 0.1459 g (5 mmol l^−1^) of the active ingredient *W*_i_(g)	theoretical content for the active ingredient at 95% (mg) *W*i (mg) ± 1.96 σ*_n_*_−1_/√*n*	[DPH·HCl] (mmol l^−1^)					
					linear regression equation at 95% confidence *Ŷ* = *a *+ Sat + *b* + Sbt[DPH·HCl] mmol l^−1^					*r* *r*^2^ *R*^2^%
					0 ml	1 ml	2 ml	3 ml	4 ml	
					0 mmol l^−1^	1 mmol l^−1^	2 mmol l^−1^	3 mmol l^−1^	4 mmol l^−1^	
					linear regression equation at 95% confidence *Ŷ* = *a*+ Sat +*b* +Sbt[DPH.HCl] mmol l^−1^ for classical method					
1	SDI Iraq 25 mg tablet	0.1255 ± 0.00224	0.73241	25 ±0.4456	135	255	390	520	650	0.9999 0.9997 99.97
					131.0000 ± 9.8076 + 129.50000 ± 4.0039 [DPH·HCl] mmol l^−1^					
					0.321 ± 0.0248 + 0.325 ± 0.0423 [DPH·HCl] mmol l^−1^					
2	Panadol night Australia 25 mg tablet	0.6746 ± 0.00408	3.9368	25 ± 0.16577	120	238	340	466	588	0.9994 0.9989 99.89
					117.60000 ± 17.6364 + 116.4000 ± 7.2000 [DPH·HCl] mmol l^−1^					
					0.213 ± 0.0248 + 0.219 ± 0.0135 [DPH·HCl] mmol l^−1^					
3	Allermin syrup SDI Iraq 10 mg	0.2 g/100 ml 6.854 mM 1.46 ml to prepare 1 mM	——–	———	142	250	430	545	690	0.9976 0.9951 99.51
					133.2000 ± 43.8862 + 139.1000 ± 17.9165 [DPH·HCl] mmol l^−1^					
					0.732 ± 0.0982 + 0.781 ± 0.0314 [DPH.HCl] mmol l^−1^

**Table 10.** Summary of results for the developed method.

no. of sample	developed method				
	classical method UV-spectrophotometric ℷ_max_ = 254 nm				
	practical concentration (mmol l^−1^) in 10 ml practical concentration (mmol l^−1^) in 100 ml practical weight of DPH·HCl in (g)	weight of DPH·HCl in each sample (g) $\bar{W}$_i(g)_ ±4.303 σ *_n_*_-1_/$\sqrt{n}$ weight of DPH·HCl in tablet $\overset{―}{W}$_i(mg)_ ±4.303σn_-1_/$\sqrt{n}$	Efficiency of determination Rec.%	individual *t*-test between claimed value & practical value ($\overset{―}{W}$_i(mg)_-*µ*) $\sqrt{n}$ /σ _n-1_	paired t –test compared between three methods
1.	1.0116 5.0579 0.1476	0.0253±0.0033	101.16	0.37879<<4.303	developed method and quoted value (reference method) $\overset{―}{X}$d = 0.03867 * σ*_*n* − 1* *_= 0.4070 0.16454<<4.303
		25.289±3.283			
	0.9870 4.9283 0.1438	0.0246±0.0024	98.57		
		24.6413±2.384			
2.	1.0103 5.0515 0.1474	0.02526±0.0035	101.03	0.3188<<4.303	
		25.258±3.482			
	0.9726 4.8630 0.1419	0.0243±0.0030	97.26		
		24.315±2.987			
3.	0.9576 6.5588 0.1914	0.0010±2.4182	95.693	/-0.80147/ <<4.303	developed method and UV-spectrophotometric method (classical method) $\overset{―}{X}$d = 0.5979 * σ*_*n* − 1* *_= 0.3725 2.7801<4.303
		9.569±2.314			
	0.9373 6.4196 187.324	0.0094±0.0030	93.66		
		9.366±2.982			

Subsequently, paired *t*-tests were performed (table 10) at a significance level of α = 0.05 (two-tailed) to compare the proposed method with both the conventional UV-spectrophotometric method and the labelled values. The results showed that there is no statistically significant difference (*p* > 0.05), indicating that the proposed method, the reference method, and the stated pharmaceutical contents are in good agreement.

Taken together, these statistical results prove the truthfulness, validity and usability of the given method of analysis for quantitatively determining DPH·HCl in various types of pharmaceutical preparations.

The statistical evaluation of DPH·HCl in the analysed pharmaceutical formulations is summarized in table 10. The accuracy of the method was determined using a single-sample *t*-test, comparing the experimentally determined values in the form of means and the labelled contents. In either instance, the *t*-values have been found to be less than the critical *t*-value (critical = 4.303, d.f. = 2, α = 0.05), therefore, no statistically significant differences were found at the 95% level. Also, to compare the results of the proposed CFIA method with the traditional UV-spectrophotometric method (254 nm), paired *t*-tests were conducted. The calculated paired *t*-values were also less than the critical threshold, which proved that the two methods of analysis show a good correlation. The close to 100% recovery percentages are also a further indication of the accuracy and trueness of the developed method of determining DPH·HCl in the pharmaceutical formulations.

**Scheme 1.**
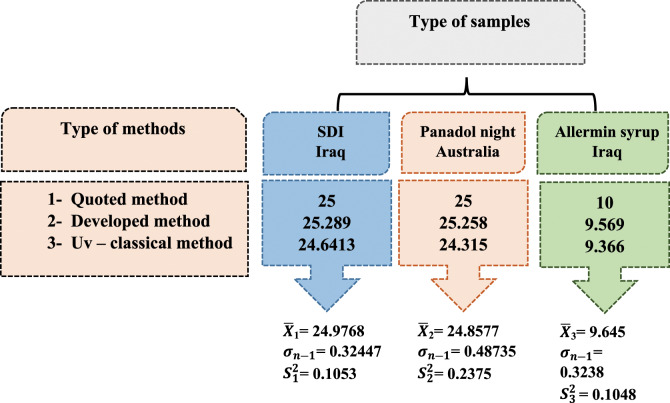
ANOVA results for three separate samples and two different values are summarized as follows: $\bar{\text{X}}$ = average of values; *σ*_*n* − 1_ = standard deviation; ${\text{S}}_{\text{i}}^{2}$ = variance.

To test the results of the outputs, a one-way ANOVA [43,44] was run at the 0.05 level of significance. The analysis was used to determine whether there were statistically significant differences between the reported values, without following the underlying operational methodologies (Scheme 1). The findings are outlined in table 11. The *F*-value calculated (*F*_cal_ = 1563.308) was significantly higher than the critical *F*-value (*F*_tab_ = 5.143, d.f. between = *k*−1, d.f. within = *N*−*K*), so the null hypothesis (H_0_) was rejected, and the alternative hypothesis (Ha) was accepted. This proves that there is statistically significant variability between the groups.

**Table 11.** ANOVA results for comparison between three different samples from three different companies.

source	sum of squares (SSq)	d.f.	mean of squares (MSq)	*F*_cal_	*F*_critical_
between group	466.5025	2	233.2512	1563.308 >>	5.143253
within group	0.895222	6	0.149204		
total	467.3977	8			

It should be noted that, on the other hand, the paired *t*-test of the CFIA and UV-spectrophotometric methods showed a *p*-value exceeding 0.05, indicating no statistically significant difference between the two methods. Thus, the one-way ANOVA identified variance within replicate groups, which expresses intra-group variability, and showed that variability does not indicate a real methodological difference between CFIA and UV measurements [45,46]. All these results point to the credibility of the CFIA method and the natural variation in the datasets of different methods; therefore, one-way ANOVA detected variance among replicate groups, reflecting intra-group variability rather than a true difference between methods.

## 5. Conclusion

In summary, the CFIA-based turbidimetric method proposed offers a sensitive, accurate and eco-friendly method for DPH·HCl measurement. The approach combines multi-angle detection with affordable WLED and solar cell technology, overcoming the main constraints of traditional spectrophotometric and single-angle turbidimetric methods and proving applicable in practice for the daily analysis of pharmaceutics. The system had higher detection sensitivity and measurement accuracy than UV-spectrophotometry, and statistical validation using *t*-tests and ANOVA demonstrated a high level of agreement between the two methods, underscoring the method's reliability and affordability. Notably, this method offers a convenient, cost-effective implementation that can be undertaken in research and industrial laboratories. Future research will aim to modify the reagents and expand the method's use on biological and clinical specimens to broaden its application in pharmaceutical and biomedical studies.

## Data accessibility

All data supporting the results of this study are available within the article.
